# Effect of cannabinoids on glutamate levels in the human brain: a systematic review and meta-analysis

**DOI:** 10.1186/s42238-025-00277-9

**Published:** 2025-04-21

**Authors:** Berzenn Urbi, Vincent Sapaen, Ian Hughes, Maame Amma Owusu, Arman Sabet, Simon A. Broadley

**Affiliations:** 1 School of Medicine, Griffith University, Brisbane, QLD Australia; 2 Research Office, Gold Coast Hospital and Health Service, Southport, QLD Australia; 3 Department of Neurology, Gold Coast Hospital and Health Service, Southport, QLD Australia

**Keywords:** Glutamate, Cannabis, Neuroimaging, Psychosis, Neurodegenerative disorder

## Abstract

Increased extracellular glutamate concentrations in the brain can cause neuronal injury. Cannabinoid use has been demonstrated to reduce extracellular glutamate levels in the brain in many animal models. However, there are no systematic reviews published evaluating the effect of cannabis on glutamate levels in the human brain. This review aimed to review studies that investigated the effect of cannabinoids on glutamate levels in the living human brain using neuroimaging methods and to provide evidence gathered from biomedical databases such as MEDLINE and EMBASE. Nine randomized controlled trials (RCTs) and ten observational studies met the eligibility criteria for this review. The articles included in the meta-analyses had a low risk of bias. Meta-analysis showed cannabis intake has no effects on the glutamate levels in human brain. However, there is limited evidence indicating that oral cannabidiol and cannabidivarin increased the glutamate/glutamine ratio in the basal ganglia while intravenous and vaped tetrahydrocannabinol increased glutamate in the basal ganglia. There is also some evidence showing oral cannabidiol increased glutamate in the hippocampus. Most of the observational studies in this review demonstrated a reduction in glutamate in the brain of chronic cannabis users. However, these findings are not definitive and will require further confirmations. This review suggests that acute cannabis administration may increase glutamate in the basal ganglia and hippocampus but not in other parts of the brain, while chronic cannabis use lead to a decrease in glutamate levels in some parts of the brain. The quality of this evidence is limited therefore further studies are needed.

**Supplementary Information:**

The online version contains supplementary material available at 10.1186/s42238-025-00277-9.

## Introduction

Glutamate is a major excitatory neurotransmitter essential for neuronal function. However, prolonged neuronal stimulation by glutamate can cause excitotoxicity (Lewerenz and Maher 2015). Glutamate excitotoxicity occurs when there is an overproduction and release of glutamate in the synaptic cleft (Dong et al. 2009). In addition to excitotoxicity, increased glutamate levels are associated with oxidative stress and inflammation, which can lead to neurodegeneration (Dong et al. 2009; Verma et al. 2022). Cannabinoids may play a role in modulating glutamate levels to prevent such damage.

There is limited direct information about the specific mechanisms of cannabidiol (CBD) and tetrahydrocannabinol (THC) on glutamate levels. However, we can infer some general effects and mechanisms from the available data: CBD and THC have distinct mechanisms of action and effects on brain function, despite their similar chemical structures (Stella 2023). THC typically boosts brain activation and blood flow, whereas CBD usually reduces them. (Gunasekera et al. 2021). This suggests that these cannabinoids may have opposing effects on neurotransmitter systems, potentially including glutamate. Interestingly, CBD has been shown to antagonize some of the effects of THC, including intoxication and sedation (Russo and Guy 2006). This antagonism could involve modulation of glutamatergic transmission, although this is not explicitly stated. The interaction between CBD and THC appears to be complex and dose-dependent, with CBD sometimes potentiating and sometimes blocking THC's effects (Karniol and Carlini 1973). Further research is needed to elucidate the precise mechanisms by which these cannabinoids affect glutamate levels in the brain.

Cannabinoid signalling can alter glutamate concentrations through a retrograde signalling mechanism involving endocannabinoids and cannabinoid receptors. This process plays a crucial role in synaptic modulation and plasticity. The primary mechanism involves the activation of postsynaptic metabotropic glutamate receptors (mGluRs), particularly mGluR1, which triggers the production and release of endocannabinoids from the postsynaptic neuron (Maejima et al. 2001). These endocannabinoids then travel backward across the synapse to activate presynaptic cannabinoid receptors, primarily CB1 receptors. Activation of CB1 receptors on presynaptic terminals leads to a reduction in neurotransmitter release, including glutamate (Maejima et al. 2001; Verma et al. 2022). However, this mechanism is spatially regulated and depends on the pattern of the synaptic activation. One study has shown that endocannabinoid regulation in cerebellar Purkinje cells of rodents only occurs when nearby synapses are activated (Marcaggi and Attwell 2005). This is due to the synaptic crosstalk detection by mGluRs. Downregulation of glutamate release by the endocannabinoid system involves an interplay between different cannabinoid receptors and the mGluRs highlighting the complex nature of synaptic regulation in the brain (Marcaggi and Attwell 2005).

Acute cannabis use has been shown to increase striatal glutamate concentrations, with the administration of THC led to dose-dependent increases in glutamate levels (Mason et al. 2021). This acute effect is thought to be associated with the psychoactive and cognitive effects of cannabis, including subjective high and decreased attention performance. The increase in striatal glutamate is also linked to alterations in dopaminergic activity and corticostriatal connectivity (Mason et al. 2021). In contrast, chronic cannabis use appears to lead to neuroadaptations that may result in tolerance to the acute effects of the drug. A study comparing occasional and chronic cannabis users found that occasional users showed significant neurometabolic alterations in the reward circuitry which includes the increase of striatal glutamate concentrations. These changes however were not seen among the chronic cannabis users (Mason et al. 2021). This suggests that for the latter, tolerance may develop as a result of long-term adaptations in the glutamatergic system. Furthermore, a systematic review investigated the effects of cannabis on brain dopamine through neuroimaging, revealed that chronic cannabis use reduced dopamine capacity and release (Sami et al. 2015). The reduction in brain dopamine reduces glutamate level and signalling (Caravaggio et al. 2016). A study by Rigucci et al. (2017) also reported lower glutamate levels in various brain regions of chronic cannabis users, particularly in the medial prefrontal cortex.

Glutamate levels in the human brain can be reliably measured using neuroimaging modalities (Cai et al. 2013). Proton magnetic resonance spectroscopy (^1^H-MRS), glutamate chemical exchange saturation transfer (GluCEST) imaging, and positron emission tomography (PET) scans provide complementary evidence for alterations in glutamate levels in various neurological conditions and physiological states. ^1^H-MRS has been used to measure glutamate concentrations in the brain, revealing alterations in conditions like schizophrenia and during sleep cycles. For instance, studies have shown reduced N-acetylaspartate (NAA) levels in the hippocampal regions of schizophrenia patients, suggesting neuronal dysfunction (Deicken et al. 2000). Additionally, ^1^H-MRS has detected overnight reductions in glutamate + glutamine (Glx) levels in healthy young adults, correlating with decreases in slow wave activity during sleep (Volk et al. 2018). GluCEST imaging offers higher spatial resolution and sensitivity compared to ^1^H-MRS, allowing for more detailed glutamate mapping. In a rat model of sepsis-induced brain injury, GluCEST values were significantly higher in sepsis-induced rats compared to controls, indicating increased glutamate levels (Lee et al. 2023). Similarly, GluCEST imaging has shown promise in detecting glutamate alterations in the spinal cord, with higher GluCEST values observed in gray matter compared to white matter (Kogan et al. 2013). Interestingly, some studies have found contradictory results between different imaging modalities. For example, while 1H-MRS showed lower glutamate + glutamine (Glx) levels in people at high-risk for psychosis compared to healthy volunteers and first-episode psychosis patients, no associations were found between glutamate metabolites and glial activation as measured by PET (Shakory et al. 2018). These imaging techniques provide valuable insights into glutamate alterations across various conditions. While ^1^H-MRS offers a well-established method for measuring glutamate, GluCEST imaging shows promise in providing higher resolution glutamate mapping. The combination of these techniques with PET scans can offer a more comprehensive understanding of glutamate dynamics in the brain and spinal cord (Poels et al. 2014).

Animal studies have demonstrated that chronic cannabis use reduces the level of glutamate in the brain (4) but some studies have also shown it may increase glutamate levels in the brain (5, 6). There is some evidence from animal studies that cannabinoids, especially THC, can prevent excessive glutamate activity in the brain. However, to date, there have been limited reviews of human studies on whether cannabis increases or decreases glutamate levels in the human brain. This review aimed to investigate the effect of cannabis on glutamate levels in the living human brain. We included studies that utilized brain imaging such as 1H magnetic resonance spectroscopy (^1^H-MRS) to measure glutamate levels in the brain.

## Methods

### Search strategy

Published and unpublished studies of the effect of cannabis on glutamate levels in the living human brain were searched on 7 th March 2024. An initial search was conducted in both MEDLINE and EMBASE using the following MeSH® terms: cannabi*, marijuana, glutamat*, and glutamic acid to develop a search strategy (Tables S1 and S2). This search strategy was applied to other databases, such as Cochrane CENTRAL, Proquest, Scopus, Web of Science, and CINAHL. ClinicalTrials.gov was also checked for any ongoing and unpublished studies. Articles were manually searched for any missing studies. The gray literature was also searched using Google.com and scholar.google.com.

### Inclusion and exclusion criteria

Randomized controlled trials and observational studies, such as open-label studies, before and after, case reports, chart reviews, and surveys that evaluated the effects of cannabis or cannabis-based treatment on glutamate levels in the living human brain, were included. We included all disease conditions and healthy populations in this review. This was done to collect as much evidence as possible. We excluded review papers but assessed these for additional relevant studies. Cannabis or cannabis-based treatment was defined as any agent that contained any cannabinoid. Tetrahydrocannabinol (THC) and cannabidiol (CBD) are among the most commonly used cannabis components. We included studies that only used brain imaging, such as ^1^H-MRS, glutamate chemical exchange saturation transfer (GluCEST), or positron emission tomography (PET), to measure glutamate. The protocol for this review was registered in PROSPERO under ID: CRD42022374016. The only change from the protocol since its registration is the explanation of the different brain imaging methods which included ^1^H-MRS, GluCEST, and PET.

This review includes all outcomes that used any measure of glutamate concentration. Commonly used measures include glutamate (Glu), glutamate and glutamine (Glx), the glutamate-glutamine to creatine ratio (Glx/Cre), and the glutamate to creatine ratio (Glu/Cre). Glutamate in the human brain can be measured using different imaging techniques. The most common imaging technique used is proton magnetic resonance spectroscopy (^1^H-MRS) (Ramadan et al. 2013; Roalf et al. 2020).

There were no restrictions on language, publication date or status. Articles in languages other than English were screened using Google Translate. The article was formally translated if it passed the screening. If the articles were deemed to cover the same data (e.g., abstract and full article), the most comprehensive or more recent article was used. Two authors independently screened and reviewed the articles (BU and VS). Any disagreements were resolved with a third author (MAO).

### Data extraction and quality assessment

The data from the included studies were extracted and assessed using an electronic form adapted from the Cochrane data collection form (Sambunjak D. 2017). The extracted data included the authors’ details, the intervention details, study population, study design, imaging methods, intervention and control group sample sizes, brain regions assessed, metabolites measured, mean and standard deviation (SD) of metabolite concentrations (as presented in studies: water scaled and tissue composition corrected “institutional concentration units”) in intervention and control groups, and t and *P* values of comparisons.

The quality of all included studies in this review was assessed using the Revised Cochrane risk-of-bias tool for randomized trials (ROB 2), with additional considerations for crossover trials (Sterne et al. 2019). The domains assessed for bias included the following: 1. Bias arising from the randomization process, 2. Bias due to deviations from intended interventions, 3. Bias due to missing outcome data; 4. Bias in measurement of the outcome, and 5. Bias in the selection of the reported result.

PRISMA 2020 and abstract checklists were constructed to provide a transparent report for this systematic review (refer to Tables S4 and S5).

### Brain regions

In the RCTs, we separated the brain areas studied into three regions: Region 1, the basal ganglia (including the striatum and caudate head); Region 2, the cortex (anterior cingulate and prefrontal); and Region 3, the hippocampus. Since different regions of the brain have different glutamate receptors (Zhou and Danbolt 2014), focusing on similar brain areas could provide more homogeneous data on the effect of cannabis on glutamate levels. Separating data by brain region was decided during the data analysis stage of this review.

### Statistical analysis

The treatment effect (effect size) was reported in all RCTs as the difference in the mean metabolite concentration between the treatment and control groups. Metabolite concentrations for each study were reported as “institutional concentration units”, which have arbitrary units based on the software and processing parameters used in ^1^H-MRS. As such, the Hedges’ standardized mean difference (H-SMD) was used as the effect size (ES). For parallel group RCTs, the standard error of the effect size SE (ES) was calculated directly from the sample sizes and standard deviations of the two groups as provided in the papers. For crossover RCTs where ES was reported as the mean of individual participant differences between treatment and placebo effects and tested using paired t-tests, the SE (ES) was estimated by dividing the ES by the t-statistic. When the t-statistic was not presented, it was estimated based on the presented *p*-values using the two-tailed inverse of the t-distribution.

Due to the differing study designs and study populations, random effects models were used for the meta-analyses. Random effects meta-analyses (DerSimonian and Laird methods) were performed using the metan command of Stata 17 (Stata Corp., College Station, Tx, USA) to obtain a pooled ES and forest plot for Glu and Glx for each brain region group. Heterogeneity was tested using the heterogeneity chi-square test and estimated using the I^2^ statistic (proportion of ES variation attributable to heterogeneity) and τ^2^ (between-study variance). The DerSimonian and Laird (D + L) method is the most commonly used method for meta-analysis but may overestimate the pooled ES when there is a small number of studies (Guolo and Varin 2017). To assess and correct for this possibility, ES was also estimated using maximum likelihood and restricted maximum likelihood methods with the mvmeta package of Stata 17 and the most consistent ES estimate presented, if different from that obtained via D + L.

## Results

### Search results and study selection

**Fig. 1:**
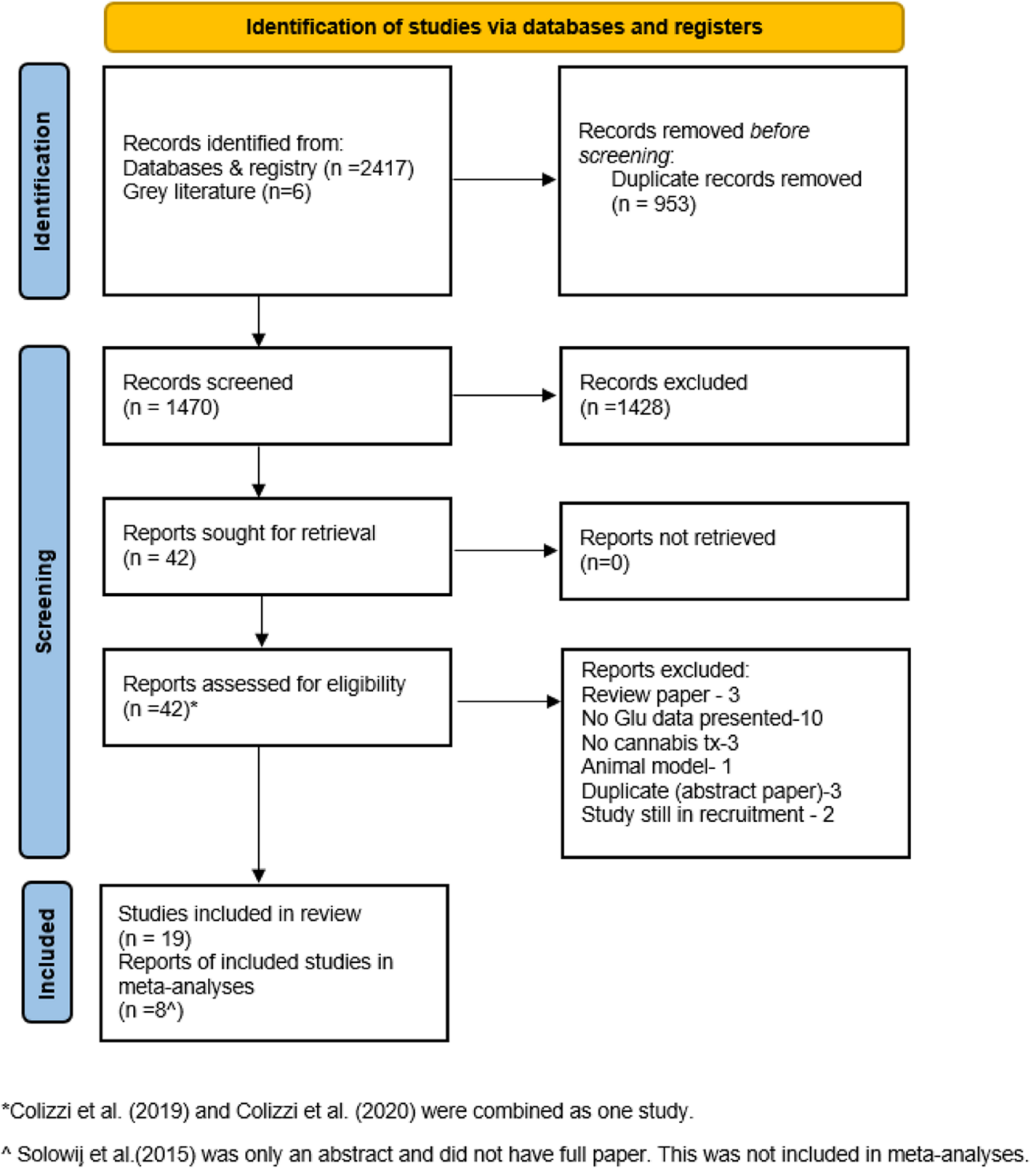
The search process and criteria for excluding articles according to the PRISMA flow diagram

The electronic searches identified 2417 journal articles. Six articles were found by searching gray literature. Authors BU and VS screened titles and abstracts, resulting in 42 eligible articles for full-text screening. There were 10 RCTs and 10 observational studies that met the eligibility criteria for this review. Twenty-two articles were excluded because they did not meet the review protocol criteria (refer to Fig. 1 for reasons for exclusion).

Articles reporting the same data, e.g., both in full articles and conference abstracts, were reviewed during the screening, and only one set of data (the full article due to the most complete dataset) was included in this review. One abstract (Colizzi et al. 2019) was combined with its full article (Colizzi et al. 2020), resulting in a total of 9 randomized studies. Additionally, one article was presented only as a conference abstract (Solowij 2015). The authors were contacted, and it was confirmed that the full study results were not published. Full data were requested, but only a poster abstract was shared, making it difficult to extract data for analysis. It was not included in any meta-analysis but still remained in this review. There were 9 randomized studies, but only 8 articles were included in the meta-analyses.

Figure 1 presents the PRISMA flow diagram of the literature search, screening, and selection of articles. The reasons for exclusion during the full-text review are also presented in this figure.

### Population characteristics

Nine RCTs investigated THC, CBD or their combination in healthy volunteers, occasional cannabis users, people with psychosis or schizophrenia, and people with autism spectrum disorder (ASD) (Bloomfield et al. 2021; Colizzi et al. 2020; Davies et al. 2023; Mason et al. 2019; O'Neill et al. 2021; Pretzsch et al. 2019a, 2019b; Solowij et al. 2015; van Boxel et al. 2023).

Ten observational studies investigated cannabis use in adolescents or cannabis users or people with schizophrenia, HIV or a history of early psychosis (Bernier et al. 2016; Blest‐Hopley et al. 2020; Chang et al. 2006; Muetzel et al. 2013; Newman et al. 2019; Prescot et al. 2011; Prescot et al. 2013; Rigucci et al. 2017; Sami et al. 2020; Subramaniam et al. 2022).

### Cannabis intervention

In RCTs, different cannabinoids and formulations were used. There are THC capsules (Bloomfield et al. 2021), intravenous (IV) (Colizzi et al. 2020) or vapor forms (Mason et al. 2019; Solowij et al. 2015). Other studies used CBD capsules (O'Neill et al. 2021), liquids (Davies et al. 2023; Pretzsch et al. 2019a, 2019b; van Boxel et al. 2023) or vapors (Solowij et al. 2015).

In observational studies, it was difficult to ascertain what form of cannabis was used. All observational studies recruited cannabis users, all of whom were compared to healthy controls, but no information on the type of cannabis consumed by the cannabis users was provided in these articles.

### Imaging modalities

All RCTs included in the meta-analysis used proton magnetic resonance spectroscopy (^1^H-MRS). Of these, four RCTs used point-resolved spectroscopy (PRESS) in quantifying glutamate levels (Bloomfield et al. 2021; Colizzi et al. 2020; Davies et al. 2023; O'Neill et al. 2021) while three RCTs used Mescher-Garwood point-resolved spectroscopy (MEGA-PRESS) (Pretzsch et al. 2019a, 2019b; van Boxel et al. 2023). Stacked stimulated echo acquisition mode (STEAM) was used by Mason et al. 2019(Mason et al. 2019), while Solowij et al. 2015 (Solowij 2015), which was not included in the meta-analysis due to no data presented, used both PRESS and MEGA-PRESS (Colizzi et al. 2020). Measurements of glutamate levels in the human brain can be reliably quantified using PRESS, MEGA-PRESS or STEAM sequence techniques (van Veenendaal et al. 2018).

### Study outcome measures

Six RCTs measured glutamate levels using Glx, two studies measured the Glx/creatine ratio (Glx/Cre), and six studies measured glutamate (Glu); however, one of these studies was presented only as an abstract (Solowij et al. 2015) (not included in the meta-analysis), while two studies measured Glu/Cre.

The detailed study characteristics of included RCTs and observational studies are presented in Tables 1 and 2.

**Table 1:** Characteristics of Randomized Clinical Trials (RCTs)

Article	Study Design	Intervention	Dose	Population	Sample Size	Duration of Treatment (Tx)	Frequency of Tx	Delivery of Tx
Bloomfield et al. 2021	COT	THC cap	15 mg	Healthy volunteers (mean age:23.5)	40	1 day	once	po
Colizzi et al. 2020	COT	THC IV	1.19 mg	Healthy volunteers (mean age: 24.4)	32	1 day	once	IV
Davies et al. 2023	RCT	CBD cap	600 mg	Clinical high risk for psychosis patients	28	1 day	once	po
Mason et al. 2019	COT	THC vapor	300ug/kg	Occasional cannabis users (mean age: 21.8)	40	1 day	One full dose or divided in 3 successive doses	Inh
O’neill et al. 2021	COT	CBD cap	600 mg	Psychotic patients (mean age: 27.73)	30	1 day	once	po
Pretzsch et al. 2019a	COT	CBD liq	600 mg	ASD patients (mean ag 29.99)	34	1 day	once	po
Pretzsch et al. 2019b	COT	CBDV liq	600 mg	ASD patients (mean age: 29.88)	34	1 day	once	po
Solowij et al. 2015	COT	THC vapor	6 mg	Human Volunteers (mean age: 21.3)	30	1 day	once	Inh
		CBD vapor	200 mg					
Van boxel et al. 2023	RCT	CBD cap	600 mg	Recent -onset Schizophrenic patients	30	28 days	daily	po

**Table 2:** Characteristics of observational studies

Article	Study Design	Intervention	Dose	Population	Sample Size	Duration of Treatment (Tx)	Frequency of Tx	Delivery of Tx
Bernier et al. 2016	CS	Cannabis*	nd	Schizophrenic pts, hx of cannabis taking > 1 year vs healthy controls	74	nd	nd	nd
Blest-Hopley et al. 2020	CS	Cannabis*	nd	Cannabis users (CU) versus non-users (NU)	43	2 years prior study participation	At least 4 days/wk for the past	nd
Chang et al. 2006	CS	Cannabis*	nd	HIV CU vs HIV NU, and Non- HIV CU and NU	HIV 42 Non-HIV 54	2.1 years	At least taking cannabis 19 days per month	nd
Muetzel et al. 2013	CS	Cannabis*	nd	Adolescent CU vs NU	48	nd	At least 5 × per week for at least one year	nd
Newman et al. 2019	CS	Cannabis*	nd	Adolescent CU vs Adolescent NU	48 (25 CU 23 NU)	nd	nd	nd
Prescot et al. 2011	CS	Cannabis*	nd	Adolescent CU vs Adolescent NU	34	Ave age of first use: 15 years old	1367 total no of cannabis smokes	Inh
Prescot et al. 2013	CS	Cannabis*	nd	Adolescent CU vs Adolescent NU	29 (16 CU vs 13 NU)	Ave age of first use: 15 years old	1124 total no of cannabis smokes	Inh
Rigucci et al. 2018	CS	Cannabis*	nd	Early Psychosis (EP) CU vs EP NU and HC	68 EP CU- 18 EP NU- 17 HC- 33	Ave age of first use: 17 years old	Either daily or weekly cannabis use	nd
Sami et al. 2020	CS	Cannabis*	nd	EP history of CU, EP with minimal CU, and HC with CU and HC w/o CU	29 EPC 25 EPMC 16 HCC 12HCMC	Ave age of first use: 16 years old	nd	nd
Subramaniam et al. 2022	CS	Cannabis*	nd	Adolescent CU vs NU	39	Ave age of regular use: 16.74 years old (mean)	At least 100 × in the last 12 months prior enrolment	nd

### Reported study quality

**Fig. 2:**
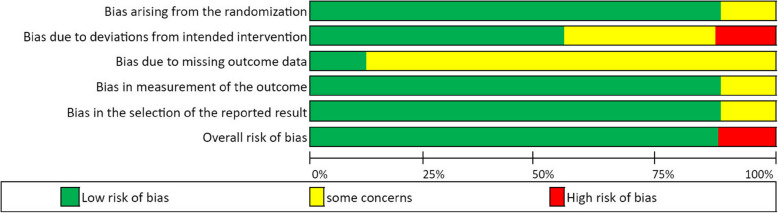
Overall risk of bias using Cochrane’s risk of bias (ROB) for randomized studies

The overall risk of bias for all randomized studies was between low risk and some risk of bias (ROB) according to Cochrane’s ROB 2 tool. The reviewers’ evaluations of ROB 2 are presented in Table S3 (Fig. 2).

### Meta-analyses of RCTs

#### Meta-analysis of Glu

Five studies measured Glu (Bloomfield et al. 2021; Colizzi et al. 2020; Davies et al. 2023; O'Neill et al. 2021; van Boxel et al. 2023). Two studies measured Glu levels in the basal ganglia (Bloomfield et al. 2021; Colizzi et al. 2020), two studies in the cortex (Colizzi et al. 2020; van Boxel et al. 2023), and three studies in the left hippocampus (Colizzi et al. 2020; Davies et al. 2023; O'Neill et al. 2021) (Tables 3 and 4).

**Table 3:** Rcts included in the meta-analysis of cortex

**Article**	**Imaging Modality and Parameters**	**OUTCOMES**				**REPORTED CONCLUSIONS**
		**Glx**	**Glu**	**Glx/Cre**	**Glu/Cre**	
Colizzi et al. 2020	1H-MRS 3 T Voxel: ACC (20 × 20x20 mm^3^) PRESS spectra analysed using LCModel v6.3 - 1L					THC did not affect glutamate levels (Glx, Glu, Glx/Cre) in the ACC
Mason et al. 2019	1H-MRS 7 T Voxel: ACC (25 × 20x17 mm^3^) STEAM spectra analysed using LCModel v6.3 - 1H					THC did not affect Glu/Cre in the ACC
Pretzsch et al. 2019a	1H-MRS 3 T Voxel: DMPFC (25 × 40x30 mm^3^) MEGA-PRESS spectra analysed using LCModel v6.3 - 1L					CBD decreased Glx level in the dmpfc
Pretzsch et al. 2019b	1H-MRS 3 T Voxel: DMPFC Midline (25 × 40x30 mm^3^) MEGA-PRESS spectra analysed using LCModel v6.3 - 1L					CBDV has no effect on Glx in the dmpfc
Van boxel et al. 2023	1H-MRS 3 T Voxel:Pre-forntal cortex (40 × 30x20 mm^3^) MEGA-PRESS spectra analysed using LCModel V6.3 - 0 A					CBD did not significantly affect glutamate levels (Glx,Glu) in the prefrontal cortex

**Table 4:** RCTs included in meta-analysis Left Hippocampus

Article	Imaging Modality and Parameters	OUTCOMES				REPORTED CONCLUSIONS
		**Glx**	**Glu**	**Glx/Cre**	**Glu/Cre**	
Colizzi et al. 2020	1H-MRS 3 T Voxel: left hippocampus (20 × 20x15 mm^3^) PRESS spectra analysed using LCModel v6.3 - 1L					THC did not affect glutamate levels (Glx, Glu, Glx/Cre) in left hippocampus
Davies et al. 2023	1H-MRS 3 T Voxel: left hippocampus (20 × 20x15 mm^3^) PRESS spectra analysed using LCModel 6.3 - 0 A					CBD did not affect glutamate levels, although CBD treated group had reduced psychotic symptoms
O'Neill et al. 2021	3 T Voxel: left hippocampus (20 × 20x15 mm^3^) PRESS spectra analysed using LCModel v6.3 - 1L					CBD increased Glu level in the hippocampus but not Glx
Solowij et al. 2015	nd					CBD increased Glu level in left hippocampus

*Legend*:  Reported increase,  Reported decrease,  No difference reported, *ACC* anterior cingulate cortex, *Cap* Capsule, *CBD* Cannabidiol, *CBDV* Cannabidivarin, *COT* Crossover (double-blind, randomized, controlled) trial, *CS* Cross sectional observational study, *CU* Cannabis user, *dmpfc* Dorsomedial prefrontal cortex, *EP* Early psychosis, *F* Female, *Glu* Glutamate, *Glu/Cre* Glutamate/creatine ratio, *Glx* Glutamine/glutamate, *HC*healthy control, *HIV* Human immunodeficiency virus, *Inh* Inhalation, *IV* Intravenous, *L* Left, *liq* Liquid, *MDMA* Methylenedioxymethamphetamine, *MEGA-PRESS* MEscher- GArwood Point RESolved Spectroscopy, *nd* no data provided, *NU* Noncannabis user, *po* per os (oral), *PRESS* Point RESolved Spectroscopy, *RCT* Randomized controlled trial (parallel group), *STEAM* Stacked stimulated echo acquisition mode, *THC* tetrahydrocannabinol, *1H-MRS* Proton magnetic resonance spectroscopy, *PRESS* Point RESolved Spectroscopy

**Fig. 3:**
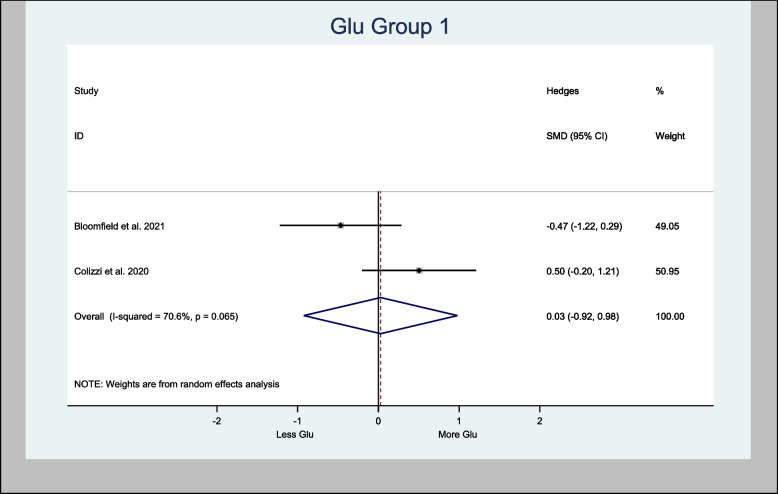
Meta- analysis of Glu (Basal Ganglia) *n* = 60; *p* = 0.954

**Fig. 4:**
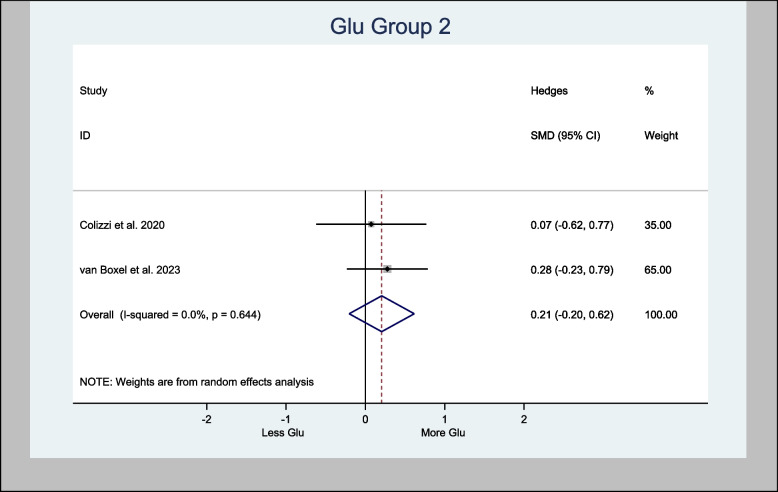
Meta- analysis of Glu (Cortex) *n* = 92; *p* = 0.326

Meta-analyses of glutamate in the basal ganglia provided an overall estimate of the Hedges’ standardized mean difference (H-SMD) of 0.03 (− 0.92, 0.98; *n* = 60; *p* = 0.954) in Glu (Fig. 3), while Glu in the cortex provided an overall estimated H-SMD of 0.21 (− 0.20, 0.62; *n* = 92; *p* = 0.326) (Fig. 4).

**Fig. 5:**
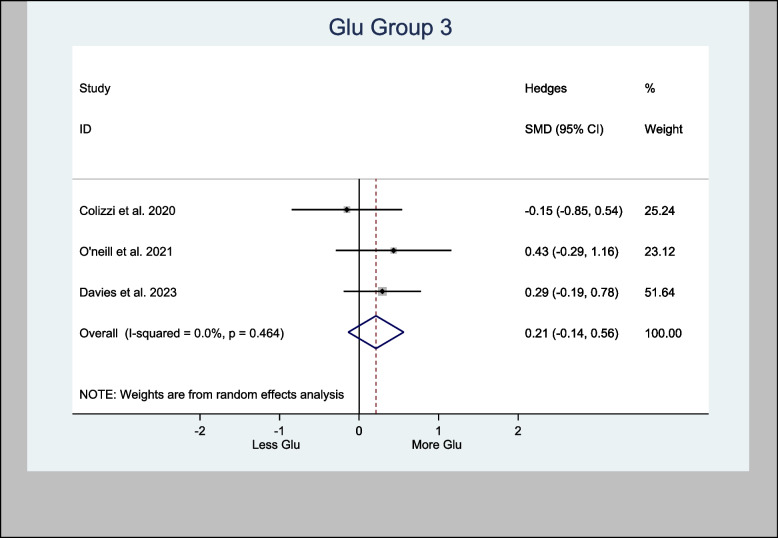
Meta- analysis of Glu (Hippocampus) *n* = 128; *p* = 0.232

**Fig. 6:**
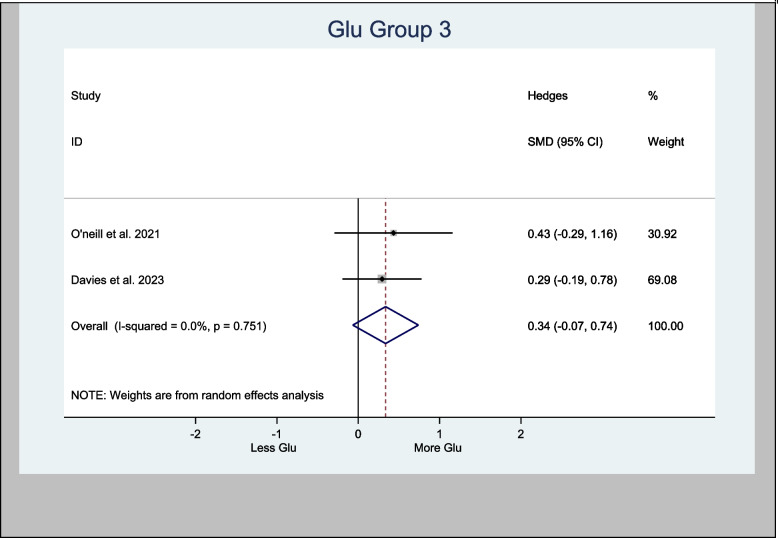
Meta- analysis of Glu (Hippocampus, CBD treatment) *n* = 96; *p* = 0.102

Glu in the left hippocampus was also meta-analyzed, with an H-SMD of 0.21 (− 0.14, 0.56; *n* = 128; *p* = 0.232) (Fig. 5). A further sub-analysis of CBD-only left hippocampus studies showed an H-SMD estimate of 0.34 (− 0.07, 0.74; *n* = 96; *p* = 0.102), indicating no difference on Glu concentration in the CBD group (Fig. 6).

#### Meta-analysis of Glx

Six of the eight RCTs included in the meta-analyses presented data on Glx (Bloomfield et al. 2021; Colizzi et al. 2020; O'Neill et al. 2021; Pretzsch et al. 2019a, 2019b; van Boxel et al. 2023).

Four studies assessed Glx in the basal ganglia (Bloomfield et al. 2021; Colizzi et al. 2020; Pretzsch et al. 2019a, 2019b), four studies measured Glx in the anterior cingulate cortex (Colizzi et al. 2020; Pretzsch et al. 2019a, 2019b; van Boxel et al. 2023), and three studies measured Glx in the left hippocampus (Colizzi et al. 2020; Davies et al. 2023; O'Neill et al. 2021).

**Fig. 7:**
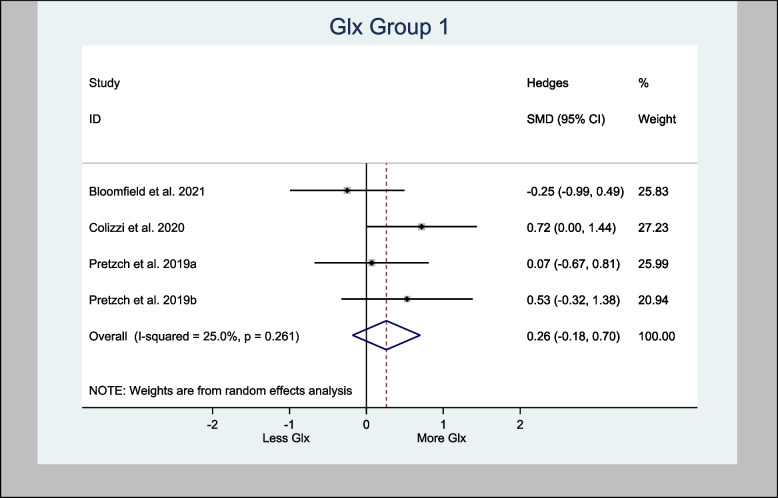
Meta- analysis of Glx (Basal Ganglia) *n* = 110; *p* = 0.246

**Fig. 8:**
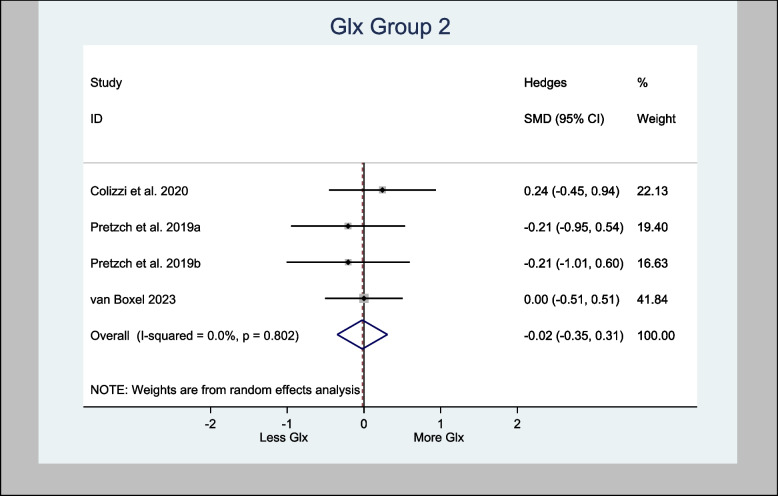
Meta- analysis of Glx (Cortex) *n* = 146; *p* = 0.900

**Fig. 9:**
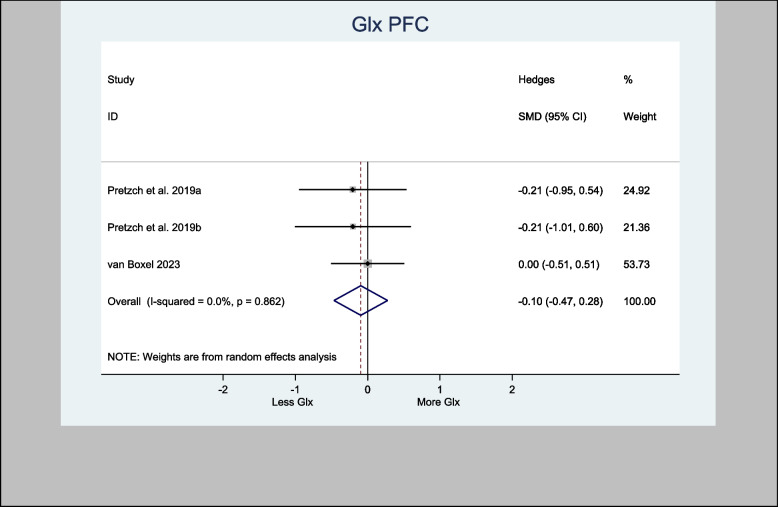
Meta- analysis of Glx (PFC) *n* = 99; *p* = 0.614

The four studies that assessed Glx in the basal ganglia specifically used the associative striatum and left caudate head. The H-SMD was 0.26 (95% CI − 0.18—0.70; *n* = 110; *p* = 0.246) for Glx, indicating no difference between the cannabinoid and placebo groups (Fig. 7). In the anterior cingulate cortex (one study) and prefrontal cortex (PFC) (three studies), the H-SMD was estimated to be − 0.02 (− 0.35, 0.31; n = 146; *p* = 0.900), indicating no difference (Fig. 8). Further sub-analysis of the three studies that measured Glx in the PFC showed an overall estimate H-SMD of − 0.10 (− 0.47, 0.28; *n* = 99; *p* = 0.614) indicating no difference (Fig. 9).

**Fig. 10:**
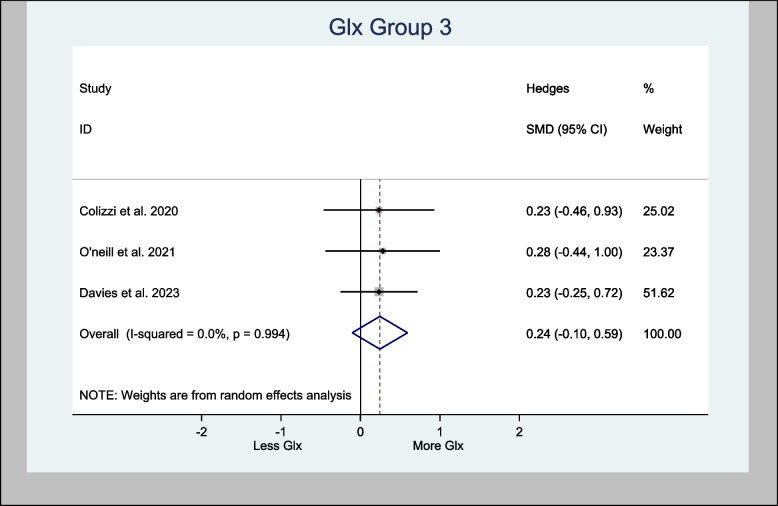
Meta- analysis of Glx (Hippocampus) *n* = 128; *p* = 0.170

The three studies that used the left hippocampus were also meta-analyzed. The analysis demonstrated an overall estimated H-SMD of 0.24 (− 0.10, 0.59; *n* = 128; *p* = 0.170), which also indicated no difference (Fig. 10).

#### Meta-analysis of the Glx/Cre ratio

**Fig. 11:**
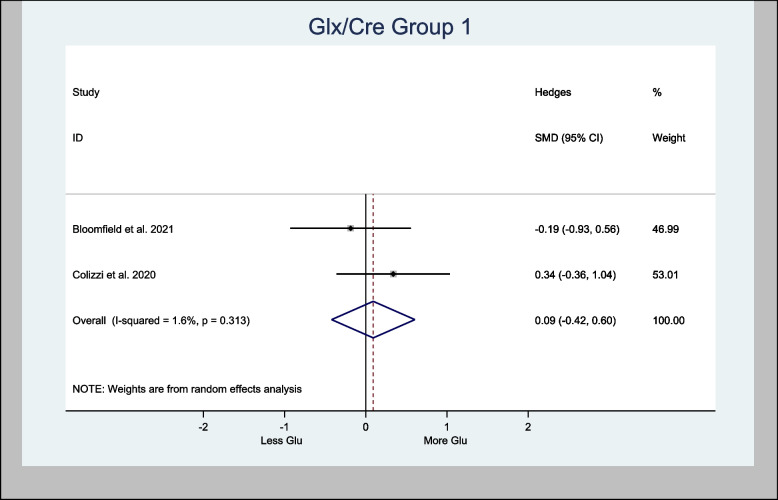
Meta- analysis of Glx/Cre (Basal Ganglia) *n* = 60; *p* = 0.726

Two studies presented data on Glx/Cre (Bloomfield et al. 2021; Colizzi et al. 2020) in the basal ganglia and were meta-analyzed. The overall estimated H-SMD of Glx/Cre was 0.09 (− 0.42, 0.61; *n* = 60; *p* = 0.726), indicating no difference (Fig. 11).

#### Meta-analysis of the Glu/Cre ratio

Three studies measured Glu/Cre in the basal ganglia brain region (Bloomfield et al. 2021; Colizzi et al. 2020; Mason et al. 2019). Two of the three studies measured Glu/Cre in the anterior cingulate cortex brain region (Colizzi et al. 2020; Mason et al. 2019).

**Fig. 12:**
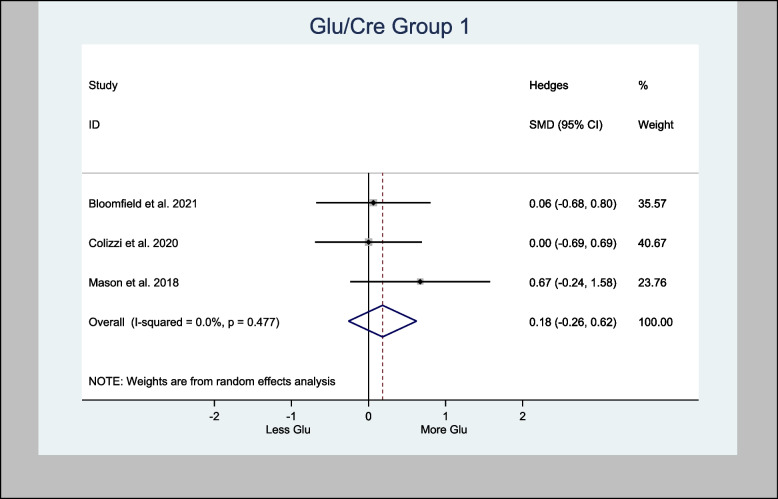
Meta- analysis of Glu/Cre (Basal Ganglia) *n* = 80; *p* = 0.421

**Fig. 13:**
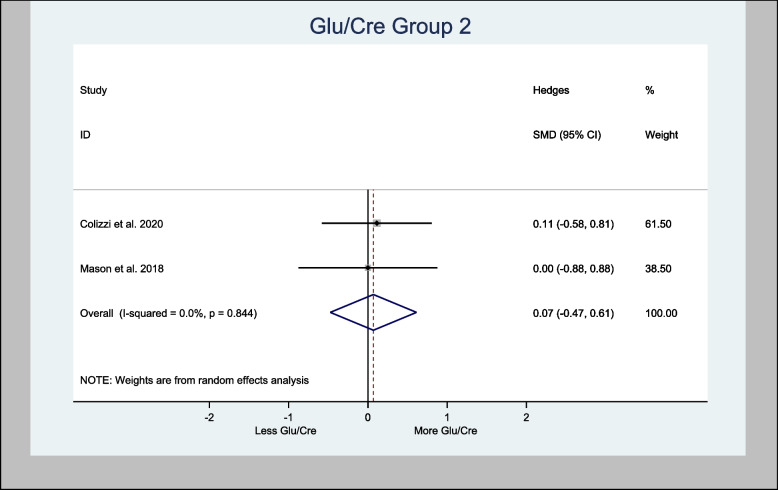
Meta-analysis of Glu/Cre (Cortex) *n* = 52; *p* = 0.804

Meta-analysis of Glu/Cre in the basal ganglia brain region demonstrated an overall estimated H-SMD of 0.18 (− 0.26, 0.62; *n* = 80; *p* = 0.421), indicating no difference (Fig. 12), while in the anterior cingulate cortex region, an overall estimated H-SMD of 0.07 (− 0.47, 0.61; *n* = 52; *p* = 0.804) also indicated no difference (Fig. 13).

#### Cannabis may increase Glx in the basal ganglia but the evidence is not definitive

An oral dose of either CBD or CBDV increased Glx levels in the basal ganglia (Pretzsch et al. 2019b, 2019a) in the ASD population. While an RCT showed that acute IV administration of THC increased Glx in the left caudate nucleus of healthy volunteers (Colizzi et al. 2020). This increase in Glx was directly associated with transient psychotomimetic effects, providing the underlying mechanism for the psychoactive effects of THC (Colizzi et al. 2020). Additionally, one RCT showed that a vaped THC for occasional cannabis users increased Glu/Cre in the basal ganglia (Mason et al. 2019). However, there are also other evidence contradicting this outcome. For instance, oral THC in healthy volunteers was associated with no changes in Glx levels in the left caudate nucleus (Bloomfield et al. 2021) or in other parts of the brain, such as the anterior cingulate cortex and hippocampus (Colizzi et al. 2020). Moreover, the meta-analysis of Glx in the basal ganglia in this review only demonstrated an H-SMD of 0.26 (95% CI − 0.18—0.70; *n* = 110; *p* = 0.246) (Fig. 7).

#### CBD may be associated with increased Glu in left hippocampus but the evidence remains inconclusive

An oral CBD capsule increased Glu in the left hippocampus of patients suffering with psychosis compared to control (O'Neill et al. 2021). A vapored CBD alone has also increased Glu in the left hippocampus of volunteers compared to control (Solowij 2015). However, other studies demonstrated the opposite. For instance, two RCTs (Colizzi et al. 2020; Davies et al. 2023) experimented on either CBD or THC showed no effects to Glu in the left hippocampus. Also, the meta-analysis of Glu effects of CBD in the left hippocampus in this review showed an H-SMD estimate of 0.34 (− 0.07, 0.74; *n* = 96; *p* = 0.102) (Fig. 6) suggesting no cannabis effect.

### Observational Studies

#### Chronic cannabis use may decrease glutamate levels

Observational studies were not meta-analyzed due to the insufficient information provided regarding the type of cannabinoids and the amount of doses used by participants in these studies. It was difficult to ascertain the quality of these studies making it difficult to proceed with any statistical analyses. What was observed from these observational studies though is that most have shown lower glutamate levels with chronic cannabis use. In chronic cannabis users, a decrease in the level of glutamate in the dorsomedial prefrontal cortex (Rigucci et al. 2017), anterior cingulate cortex (ACC) of adolescents (Prescot et al. 2011; Prescot et al. 2013), striatum (Muetzel et al. 2013; Newman et al. 2019) and basal ganglia (Chang et al. 2006) has been reported. In contrast, other studies have shown that chronic cannabis use does not affect the level of glutamate in the ACC, caudate, or hippocampal region of the brain (Sami et al. 2020; Subramaniam et al. 2022; Blest‐Hopley et al. 2020).

## Discussion

The review shows that cannabis did not affect the glutamate levels in the living human brain. However, an acute cannabis intake may be associated with an increase of glutamate levels in the basal ganglia and hippocampus but noting that the evidence is often contradictory hence inconclusive. Chronic consumption of cannabis, on the other hand, may eventually reduce glutamate levels in the brain but evidence are mostly from observational studies. Studies included in this review are limited by the varying experimental study designs and methods. We have noted our observations from this review, which require further investigation to better understand the relationship between cannabinoids and glutamate.

### Variations in study design

The following considerations should be taken into account when interpreting the results from the included studies. There are many differences in how each study conducted its experiments, and these differences are discussed below.

#### Types of participants

The intent of this review is to comprehensively investigate the effects of cannabis on glutamate levels in different population groups. This resulted in included studies reporting cannabis treatment for participants with a history of psychosis (O'Neill et al. 2021; Davies et al. 2023) or schizophrenia (van Boxel et al. 2023), patients with ASD (Pretzsch et al. 2019a, 2019b), or those who had occasionally used cannabis (Mason et al. 2019). All these factors may have affected the level of glutamate.

#### Age

There is a reduction in glutamate in the motor cortex with neuronal loss as people age (Kaiser et al. 2005). An age-related reduction in glutamate in the anterior cingulate cortex has been noted in women (Hädel et al. 2013). The eight studies included in this review recruited participants aged 21–30 years, with 25 as the mean age. Although the evidence showed a reduction in glutamate in older people, participants in the included studies were younger, hence representing a good sample for studying the effects of cannabis on glutamate.

#### Routes of administration

The outcome may have been affected by the different routes of cannabis administration in each study (oral, IV and inhalation) as well as the cannabinoids used (THC, CBD, CBDV). After inhalation, regardless of sex, the THC is greater in the brain than after injection (Baglot et al. 2021).

#### Imaging protocol used in each study

The type of imaging used in each study may also have affected the outcome. Proton magnetic resonance spectroscopy (^1^H-MRS) has been used to noninvasively measure brain metabolites, including glutamate. The ^1^H-MRS has shown high reliability in measuring glutamate in the brain (Marsman et al. 2017; Liu et al. 2017). However, because glutamate has a chemical structure that is very similar to that of glutamine, it is difficult to distinguish between the two (Ramadan et al. 2013). Human magnetic resonance imaging magnetic fields can also affect glutamate measurement. 3 Tesla (3 T) has been traditionally used to measure brain metabolites including glutamate but ultra-high imaging resolution such as 7 Tesla (7 T) provides better image quality demonstrated by higher signal-to-noise and contrast-to-noise ratios (Okada et al. 2022). All RCTs used 3 T magnetic field except for Mason et al. (2019) who used 7 T. Additionally, quantification techniques in imaging may also affect glutamate measurement using PRESS, MEGA-PRESS or STEAM sequence techniques (van Veenendaal et al. 2018), although these techniques have been demonstrated to be similar in measuring glutamate (van Veenendaal et al. 2018; Gonen et al. 2020). Nonetheless, ^1^H-MRS provides only a bulk assessment of glutamate metabolites due to the poor spatial resolution of ^1^H-MRS used in these studies (Pretzsch et al. 2019a, 2019b), limiting measurements of glutamate metabolites between the intra- and extracellular levels.

This review did not find any studies that utilized GluCEST or PET.

The majority of studies measuring metabolites in human brain utilized 1H-MRS. While GluCEST, a newer imaging method which was recently introduced in 2012 (Cai et al. 2013), are limited due to various imaging protocols published in the literature (Cember et al. 2023). PET on the other hand is a better imaging tool to visualize and measure glutamate levels in the human brain. However, it is also of limited utility due to radiation exposure for participants and the complex imaging processes required (Chen et al. 2016).

#### Different brain regions

Different parts of the brain have different concentrations of glutamate, (Grimm et al. 2012; Basu et al. 2022) and some parts are more sensitive to cannabis (Yücel et al. 2016). The hippocampus can be damaged by exposure to THC (Burggren et al. 2019; Yücel et al. 2016) with a consequent decrease in glutamate concentration, whereas CBD exposure has neuroprotective effects (Yücel et al. 2016).

Compared with that in white matter, the glutamate concentration in gray matter is 40% greater, with the amygdala having the highest concentration (Cai et al. 2013). In subcortical regions, gray matter has significantly greater glutamate levels than white matter (Cai et al. 2013). In a GluCEST study of nonhuman primates, there were increased glutamate levels in the nucleus accumbens, septum, basal forebrain, and cortical areas (Garin et al. 2022). Hence, in this study, all the results were meta-analyzed by brain region for better accuracy of the results and interpretation.

#### Exposure to cannabis

The RCTs included in the meta-analysis had variations on their requirement for previous exposure to cannabis for participants. Some studies required that participants abstain from using cannabis for at least a month before their enrolment. (Pretzsch et al. 2019a, 2019b; van Boxel et al. 2023), for the past 6 months (Colizzi et al. 2020), or the past 96 h (Davies et al. 2023). Other RCTs included participants who were occasional cannabis where users averaging 5 cannabis intake per month (Mason et al. 2019). Also, one RCT required participants to have at least one cannabis exposure, although it was undefined, (Bloomfield et al. 2021) while another RCT did not exclude participants with previous cannabis exposure (O'Neill et al. 2021).

### Acute versus chronic cannabis use

Some RCTs investigated the acute effects of cannabinoids and demonstrated that CBD and THC may increase glutamate levels in the basal ganglia (Pretzsch et al. 2019a, 2019b) and striatum (Colizzi et al. 2020; Mason et al. 2019) and hippocampus (O'Neill et al. 2021; Solowij 2015) despite other RCTs demonstrating no glutamate effects of cannabis in the striatum, cortex and hippocampus. On the other hand, most observational studies in this review demonstrated chronic cannabis exposure is correlated with reduced glutamate level in the basal ganglia (Chang et al. 2006; Newman et al. 2019) and cortex (Prescot et al. 2011; Prescot et al. 2013; Rigucci et al. 2017), and is consistent with the findings of a prior review (Colizzi et al. 2016). However, these observations are inconclusive and further studies are required.

Chronic cannabis intake was associated with decreased glutamate which could be due to desensitization and tolerance to long-term cannabis exposure (Sami et al. 2020). Animal and human studies have demonstrated that cannabis receptors undergo downregulation and desensitization in both cortical and subcortical regions of the brain after chronic cannabis intake (Breivogel et al. 1999; Sim-Selley 2003; Sim et al. 1996; D'Souza et al. 2016), resulting in cannabis tolerance. Cannabis tolerance occurs when regular cannabis users have reduced behavioral and physiological effects after repeated cannabis exposure due to cannabinoid receptor downregulation and a reduction in interactions between ligands and receptors (Ameri 1999). In fact, regular cannabis users are highly likely to develop tolerance to cannabis effects (Colizzi and Bhattacharyya 2018b). However, desensitization and tolerance can be reversed when cannabis use ceases (D'Souza et al. 2016), suggesting that neuro-adaptability changes do occur (González et al. 2005).

Any conclusions drawn from the studies in this review need to consider the previous cannabis exposure of participants, as cannabis-naïve users have a greater tendency toward side effects such as anxiety and psychosis (Hall 2009), as well as an individual’s sensitivity to cannabis and its effects (Bhattacharyya et al. 2012). Some RCTs included in the meta-analysis required participants to abstain from cannabis for periods ranging from 3 to 90 days, while other studies did not clearly outline this requirement. This information should be considered when interpreting these results.

## Conclusion

This review revealed that cannabis did not have any effects on glutamate levels in the living human brain. There is limited evidence to suggest that oral CBD may increase Glx in the basal ganglia and hippocampus, while there is also some evidence that IV THC may elevate Glx in the left caudate nucleus. Additionally, vaped THC appears to increase Glx in the striatum. On the other hand, long-term cannabis use decreased overall glutamate levels in most areas of the brain. However, these findings are not confirmatory and require further investigation. Other studies within this review also demonstrated no glutamate effect from cannabis intake across different human brain regions. Variations in study design, such as imaging modality and parameters, prior cannabis exposure of participants, and cannabis products used, were noted among the studies. More research is needed to determine the true effect of cannabis on glutamate levels in the living human brain.

### Implications for clinical practice and future research

This study contributes to understanding the neurochemical effects and therapeutic implications of cannabinoids in the brain. By examining how cannabinoids affect the brain, potential therapies for various neurological conditions linked to imbalances in glutamate levels can be investigated. Furthermore, understanding how individuals react differently to glutamate depending on age, sex, and duration of cannabis use, among other factors, may enable clinicians to customize treatments according to each patient’s needs. The findings of this review can assist in shaping future clinical trials, particularly regarding selection of study participants, route of administration, and duration of cannabis treatment. As the relationship between cannabis and glutamate levels remains uncertain, further investigation is warranted in disorders linked to elevated glutamate in the brain. A well-structured clinical trial, guided by the insights from this review, would be a valuable approach to exploring this potential.

## Supplementary Information

Supplementary Material 1

Supplementary Material 2

Supplementary Material 3

Supplementary Material 4

## Data Availability

The data used for the meta-analyses are available from a public, open access repository site. Figshare: 10.6084/m9.figshare.25762611.

## Abbreviations

CBD: Cannabidiol
CBDV: Cannabidivarin
Glu: Glutamate
Glx: Glutamate and glutamine ratio
Glu/Cre: Glutamate and creatine ratio
Glx/Cre: Glutamate-glutamine and creatine ratio
^1^H-MRS: ^1^H magnetic resonance spectroscopy
RCT: Randomized controlled studies
THC: Tetrahydrocannabinol
