# Supplementary material for: Effect of cannabinoids on glutamate levels in the human brain: a systematic review and meta-analysis
Source: J Cannabis Res. 2025 Apr 21;7:21. doi: 10.1186/s42238-025-00277-9 (PMC12010670; doi:10.1186/s42238-025-00277-9)
Supplement: Supplementary file 1 — Supplementary Material 1 [file 42238_2025_277_MOESM1_ESM.docx]

**Table S2. EMBASE Search Strategy**

**EMBASE (Embase.com)** **07/03/2024**

| **No.** | **Query** | **Results** |
| --- | --- | --- |
| #30 | #3 AND #7 AND #29 | 379 |
| #29 | #23 NOT #28 | 10082942 |
| #28 | #24 OR #27 | 5083997 |
| #27 | #25 NOT #26 | 4217377 |
| #26 | 'human'/de OR 'normal human'/de | 27312210 |
| #25 | 'animal experiment'/de OR 'animal'/de | 5240495 |
| #24 | rat:ti OR rats:ti OR cow:ti OR cows:ti OR bovine:ti OR chicken*:ti OR horse:ti OR horses:ti OR mice:ti OR mouse:ti OR pig:ti OR pigs:ti OR piglet*:ti OR animal*:ti | 2263276 |
| #23 | #8 OR #9 OR #10 OR #11 OR #12 OR #13 OR #14 OR #15 OR #16 OR #17 OR #18 OR #19 OR #20 OR #21 OR #22 | 10684589 |
| #22 | 'case control':ti, ab | 210258 |
| #21 | (case NEXT/1 (control* OR series OR report* OR study OR studies)):ti, ab | 1189507 |
| #20 | survey*:ti, ab OR questionnaire*:ti, ab OR 'cross section*':ti, ab | 2373094 |
| #19 | randomly:ti, ab OR randomized:ti, ab OR placebo:ti, ab | 1615859 |
| #18 | random*:ti OR rct:ti | 410423 |
| #17 | 'quasi experiment*':ti, ab OR quasiexperiment*:ti, ab OR 'quasi random*':ti, ab OR quasirandom*:ti, ab OR 'quasi control*':ti, ab OR (((quasi* OR experimental) NEXT/2 (method* OR study OR studies OR trial OR design*)):ti, ab) | 253356 |
| #16 | (pre NEXT/3 post):ti, ab | 207638 |
| #15 | 'pre test':ti, ab OR pretest:ti, ab OR preintervention:ti, ab OR 'pre intervention':ti, ab OR 'post test':ti, ab OR posttest:ti, ab OR postintervention:ti, ab OR 'post intervention':ti, ab | 112939 |
| #14 | ((single OR double* OR triple* OR treb*) NEXT/1 (blind* OR mask*)):ti, ab | 289411 |
| #13 | prospective:ti, ab | 1139423 |
| #12 | longitudinal:ti, ab | 460199 |
| #11 | (observational NEXT/1 (study OR studies)):ti, ab | 268840 |
| #10 | (('follow up' OR 'follow-up') NEXT/1 (study OR studies OR assessment)):ti, ab | 84630 |
| #9 | (cohort NEXT/1 (study OR studies OR analys*)):ti, ab | 509368 |
| #8 | 'randomization'/de OR 'clinical trial'/de OR 'controlled clinical trial'/de OR 'randomized controlled trial'/de OR 'single blind procedure'/de OR 'double blind procedure'/de OR 'control group'/de OR 'clinical study'/de OR 'cohort analysis'/de OR 'case control study'/de OR 'pilot study'/de OR 'observational study'/de OR 'evaluation study'/de OR 'program evaluation'/de OR 'comparative study'/de OR 'comparative effectiveness'/de OR 'multicenter study'/de OR 'follow up'/de OR 'prospective study'/de OR 'quasi experimental study'/de OR 'cross-sectional study'/de OR 'questionnaire'/de | 7623833 |
| #7 | #4 OR #5 OR #6 | 130861 |
| #6 | cannabis:ti, ab OR marijuana:ti, ab OR cannabin*:ti, ab OR cannabid*:ti, ab OR phytocannabin*:ti, ab OR endocannabin*:ti, ab OR tetrahydrocannabin*:ti, ab OR thc:ti, ab OR cbd:ti, ab | 104402 |
| #5 | 'cannabinoid'/exp | 93404 |
| #4 | 'cannabis'/de OR 'cannabis (genus)'/exp OR 'cannabaceae'/de | 49320 |
| #3 | #1 OR #2 | 222446 |
| #2 | glutamat*:ti, ab OR 'l glutamat*':ti, ab OR 'd glutamat*':ti, ab OR 'glutamic acid':ti, ab OR 'l-glutamic acid':ti, ab | 185034 |
| #1 | 'glutamic acid'/de OR 'glutamic acid derivative'/de | 117145 |
