# Supplementary material for: Effect of cannabinoids on glutamate levels in the human brain: a systematic review and meta-analysis
Source: J Cannabis Res. 2025 Apr 21;7:21. doi: 10.1186/s42238-025-00277-9 (PMC12010670; doi:10.1186/s42238-025-00277-9)
Supplement: Supplementary file 2 — Supplementary Material 2 [file 42238_2025_277_MOESM2_ESM.docx]

**Table S3 Risk of bias**

| Bloomfield et al. 2021 | | |
| --- | --- | --- |
| *Bias* | ***Reviewers’ judgment*** | ***Support for judgment*** |
| *Bias arising from randomization* | Low risk | Although the article mentioned about using randomization, no specific details on how randomization was generated. No information about protecting allocation concealment. No issues with baseline differences between groups |
| *Bias due to deviations from intended intervention* | Low risk | This study was randomized controlled double-blind crossover design. All assessors and participants were blinded to treatment allocation. There is one week washover period is sufficient for this trial. |
| *Bias due to missing outcome data* | Some concerns | 6 participants were excluded due to poor MRI data quality, however, unclear what treatment group these 6 participants were. Difficult to ascertain the direction of bias due to missing outcome data |
| *Bias in measurement of the outcome* | Low risk | Blinding of participants and assessors. Assessment of the outcome unlikely influenced by knowledge of intervention received. |
| *Bias in the selection of the reported result* | Low risk | Statistical method appropriate for the study. The result unlikely to have been selected on the basis of the results from multiple eligible outcome measurements and multiple eligible analyses of data. |
| *Overall risk of bias* | Low risk |  |

| Colizzi et al. 2022 | | |
| --- | --- | --- |
| *Bias* | ***Reviewers’ judgment*** | ***Support for judgment*** |
| *Bias arising from randomization* | Low risk | Although the article mentioned about using randomization, no specific details on how randomization was generated. No information about protecting allocation concealment. Not enough information on baseline characteristics of participants presented. |
| *Bias due to deviations from intended intervention* | Some concerns | This study was randomized controlled double-blind crossover design. All assessors and participants were blinded to treatment allocation. 2-week washout period is appropriate for this study. Participants might have been aware they were on THC due to psychoactive side effects. |
| *Bias due to missing outcome data* | Some concerns | Difficult to ascertain if any participant were excluded, or if all participants were included in analysis. Not enough information provided. |
| *Bias in measurement of the outcome* | Low risk | Blinding of participants and assessors. Assessment of the outcome unlikely influenced by knowledge of intervention received. |
| *Bias in the selection of the reported result* | Low risk | Statistical method appropriate for the study. The result unlikely to have been selected on the basis of the results from multiple eligible outcome measurements and multiple eligible analyses of data. |
| *Overall risk of bias* | Low risk |  |

| Davies et al. 2023 | | |
| --- | --- | --- |
| *Bias* | ***Reviewers’ judgment*** | ***Support for judgment*** |
| *Bias arising from randomization* | Low risk | Although the article mentioned about using randomization, no specific details on how randomization was generated. No information about protecting allocation concealment. No issues with baseline differences between groups |
| *Bias due to deviations from intended intervention* | Low risk | This study was randomized double-blind. All assessors and participants were blinded to treatment allocation. |
| *Bias due to missing outcome data* | Some concerns | CBF data were missing for several CHF subjects that may have impacted on the statistical power of the combined glutamate-CBF analyses Difficult to ascertain the direction of bias due to missing outcome data |
| *Bias in measurement of the outcome* | Low risk | Blinding of participants and assessors. Assessment of the outcome unlikely influenced by knowledge of intervention received. |
| *Bias in the selection of the reported result* | Low risk | Statistical method appropriate for the study. The result unlikely to have been selected on the basis of the results from multiple eligible outcome measurements and multiple eligible analyses of data. |
| *Overall risk of bias* | Low risk |  |

| Mason et al. 2019 | | |
| --- | --- | --- |
| *Bias* | ***Reviewers’ judgment*** | ***Support for judgment*** |
| *Bias arising from randomization* | Low risk | Although the article mentioned about using randomization, no specific details on how randomization was generated. No information about protecting allocation concealment. Some information on baseline characteristics of participants presented. |
| *Bias due to deviations from intended intervention* | Some concerns | This study was randomized controlled double-blind crossover design. All assessors and participants were blinded to treatment allocation. 1-week washout period is appropriate for this study. Participants might have been aware they were on THC due to psychoactive side effects. |
| *Bias due to missing outcome data* | Some concerns | Difficult to ascertain if any participant were excluded, or if all participants were included in analysis. Not enough information provided. |
| *Bias in measurement of the outcome* | Low risk | Blinding of participants and assessors. Assessment of the outcome unlikely influenced by knowledge of intervention received. |
| *Bias in the selection of the reported result* | Low risk | Statistical method appropriate for the study. The result unlikely to have been selected on the basis of the results from multiple eligible outcome measurements and multiple eligible analyses of data. |
| *Overall risk of bias* | Low risk |  |

| O’Neill et al. 2021 | | |
| --- | --- | --- |
| *Bias* | ***Reviewers’ judgment*** | ***Support for judgment*** |
| *Bias arising from randomization* | Low risk | Randomization sequence was described and appropriate for the study. No information about protecting allocation concealment. Some information on baseline characteristics of participants presented. |
| *Bias due to deviations from intended intervention* | Low risk | This study was randomized controlled double-blind crossover design. All assessors and participants were blinded to treatment allocation. 1-week washout period is appropriate for this study. |
| *Bias due to missing outcome data* | Some concerns | Difficult to ascertain if any participant were excluded, or if all participants were included in analysis. Not enough information provided. |
| *Bias in measurement of the outcome* | Low risk | Blinding of participants and assessors. Assessment of the outcome unlikely influenced by knowledge of intervention received. |
| *Bias in the selection of the reported result* | Low risk | Statistical method appropriate for the study. The result unlikely to have been selected on the basis of the results from multiple eligible outcome measurements and multiple eligible analyses of data. |
| *Overall risk of bias* | Low risk |  |

| Pretzsch et al. 2019a | | |
| --- | --- | --- |
| *Bias* | ***Reviewers’ judgment*** | ***Support for judgment*** |
| *Bias arising from randomization* | Low risk | Randomization sequence was described and appropriate for the study. Information about protecting allocation concealment described. Some information on baseline characteristics of participants presented. |
| *Bias due to deviations from intended intervention* | Low risk | This study was randomized controlled double-blind crossover design. All assessors and participants were blinded to treatment allocation. 13-day washout period is appropriate for this study. |
| *Bias due to missing outcome data* | Some concerns | Some participants were not included in final analyses due to poor data quality. Difficult to ascertain direction of bias due to insufficient data. |
| *Bias in measurement of the outcome* | Low risk | Blinding of participants and assessors. Assessment of the outcome unlikely influenced by knowledge of intervention received. |
| *Bias in the selection of the reported result* | Low risk | Statistical method appropriate for the study. The result unlikely to have been selected on the basis of the results from multiple eligible outcome measurements and multiple eligible analyses of data. |
| *Overall risk of bias* | Low risk |  |

| Pretzsch et al. 2019b | | |
| --- | --- | --- |
| *Bias* | ***Reviewers’ judgment*** | ***Support for judgment*** |
| *Bias arising from randomization* | Low risk | Randomization sequence was described and appropriate for the study. Information about protecting allocation concealment described. Some information on baseline characteristics of participants presented. |
| *Bias due to deviations from intended intervention* | Low risk | This study was randomized controlled double-blind crossover design. All assessors and participants were blinded to treatment allocation. 13-day washout period is appropriate for this study. |
| *Bias due to missing outcome data* | Some concerns | Some participants were not included in final analyses due to data quality. Difficult to ascertain direction of bias due to insufficient data. |
| *Bias in measurement of the outcome* | Low risk | Blinding of participants and assessors. Assessment of the outcome unlikely influenced by knowledge of intervention received. |
| *Bias in the selection of the reported result* | Low risk | Statistical method appropriate for the study. The result unlikely to have been selected on the basis of the results from multiple eligible outcome measurements and multiple eligible analyses of data. |
| *Overall risk of bias* | Low risk |  |

| Solowij et al. nd | | |
| --- | --- | --- |
| *Bias* | ***Reviewers’ judgment*** | ***Support for judgment*** |
| *Bias arising from randomization* | Some concerns | Nil information on randomization sequence, allocation concealment and baseline difference between groups described in the poster abstract supplied by author. |
| *Bias due to deviations from intended intervention* | High risk | Nil information about protection of blinding and nonadherence of participants in the poster abstract supplied by the author. |
| *Bias due to missing outcome data* | Some concerns | Participants’ data presented. Not enough information presented. |
| *Bias in measurement of the outcome* | Some concerns | Outcome tool was deemed appropriate for the study aim. Not enough information. |
| *Bias in the selection of the reported result* | Some concerns | Statistical method appropriate for the study. Although not enough information presented to determine if all outcomes were reported. |
| *Overall risk of bias* | High risk | The author provided abstract poster to the review team. However, not enough information can be gathered from the abstract to fully assess biases, hence, high risk of bias. |

| Van Boxel et al. 2023 | | |
| --- | --- | --- |
| *Bias* | ***Reviewers’ judgment*** | ***Support for judgment*** |
| *Bias arising from randomization* | Low risk | Although the article mentioned about using randomization, no specific details on how randomization was generated. No information about protecting allocation concealment. No issues with baseline differences between groups |
| *Bias due to deviations from intended intervention* | Low risk | This study was randomized double-blind. All assessors and participants were blinded to treatment allocation. |
| *Bias due to missing outcome data* | Low risk | Data for all randomized participants are presented. |
| *Bias in measurement of the outcome* | Low risk | Blinding of participants and assessors. Assessment of the outcome unlikely influenced by knowledge of intervention received. |
| *Bias in the selection of the reported result* | Low risk | Statistical method appropriate for the study. The result unlikely to have been selected on the basis of the results from multiple eligible outcome measurements and multiple eligible analyses of data. |
| *Overall risk of bias* | Low risk |  |
